# Ab Initio Modeling of MultiWall: A General Algorithm First Applied to Carbon Nanotubes

**DOI:** 10.1021/acs.jpca.1c01682

**Published:** 2021-04-28

**Authors:** Naiara Leticia Marana, Yves Noel, Julio Ricardo Sambrano, Chiara Ribaldone, Silvia Casassa

**Affiliations:** † Modeling and Molecular Simulation Group-CDMF, São Paulo State University, UNESP, 17033-360 Bauru, SP, Brazil; ‡ Institut des Sciences de la Terre Paris (iSTeP), Sorbonne Université, 75006 Paris, France; § Theoretical Group of Chemistry, Chemistry Department I.F.M., Torino University, Torino 10124, Italy

## Abstract

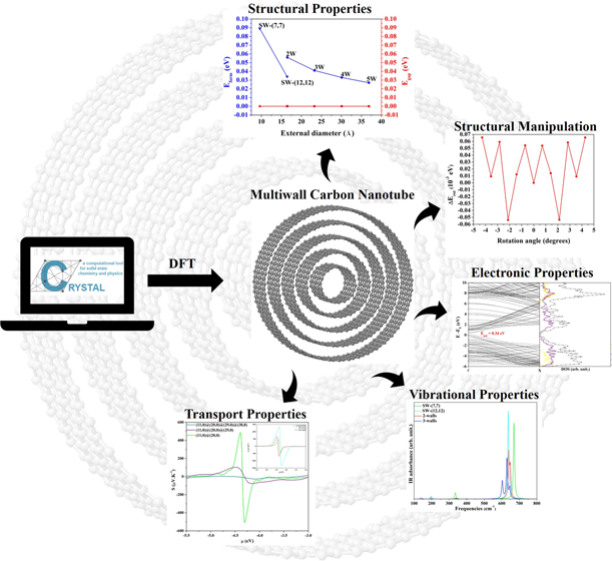

A general, versatile and automated computational algorithm to design any type of multiwall nanotubes of any chiralities is presented for the first time. It can be applied to rolling up surfaces obtained from cubic, hexagonal, and orthorhombic lattices. Full exploitation of the helical symmetry permits a drastic reduction of the computational cost and therefore opens to the study of realistic systems. As a test case, the structural, electronic, mechanical, and transport properties of multiwall carbon nanotubes (MWCNT) are calculated using a density functional theory approach, and results are compared with those of the corresponding layered (graphene-like) precursors. The interaction between layers has a general minimum for the inter-wall distance of ≈3.4 Å, in good agreement with experimental and computed optimal distances in graphene sheets. The metallic armchair and semiconductor zigzag MWCNT are almost isoenergetic and their stability increases as the number of walls increases. The vibrational fingerprint provides a reliable tool to identify the chirality and the thickness of the nanostructures. Finally, some promising thermoelectric features of the semiconductor MWCNT are reproduced and discussed.

## 1 Introduction

In the last decades, with the advancement of nanotechnology, different structures emerged, characterized by promising and appealing properties. Such structures boosted the progress in material sciences and fostered not only the research but also the application of nanomaterials in the most diverse areas, from medicine to electronic devices.^1^

Alongside the synthesis and the experimental characterization of such materials, the development of reliable computational tools can be a good way to assess their fundamental properties and explore the effects of chiralities, thickness, and doping process to obtain a preliminary screening of potentially interesting systems for technological and scientific applications.

With this goal, in 2010, Noel and co-workers^2^ implemented in the CRYSTAL program an original algorithm that fully exploits the helical symmetry in a periodic contest and allows the modeling and simulation of single-wall nanotubes (SWNTs).^3^ In particular, by defining very few input parameters, it is possible to design nanotubes of any diameter and chirality, starting from slabs and/or bulk of different materials. The ability of the code to deal with the ground state properties, its accuracy, and generality has been widely demonstrated.^4−7^ Moreover, more recently, ZnO/AlN/GaN nanotubes have been fully characterized with respect to their reactivity toward small molecules of catalytic interest.^8−10^ Unfortunately, this method only allowed single-wall modeling. Double-wall nanotubes have been conveniently conceived by wrapping a double-layer slab^11,12^ but in this kind of strategy, the structure is subjected to a strain due to the wrapping procedure that elongates the bonds and deforms the bond angles. This deformation effect can lead to the calculation of incorrect or unrealistic properties. Furthermore, the extension to systems with more than two walls is neither direct nor general. As far as we know, few studies have been done on multiwall (MW) systems,^13,14^ perhaps for this very reason.

Experimentally, MW nanotubes are routinely synthesized and studied. On the one hand, a greater thickness can be deliberately sought to increase the strength of the material and improve its performance. On the other hand, it can be difficult to control the wall growth during the synthesis process so that many nanotubes may have a thickness of a few nanometers, which corresponds to some walls.

To fill the gap between the theory and experiment and to turn the research in nanotubes more effective, it would be desirable to have a tool to design, manipulate and computed MW nanotubes. Hence, the above algorithm was extended by Noel, based on an original scheme proposed by Dovesi, to address *M*-wall nanotubes (*M* ≥ 2) obtained by wrapping any type of layered material in different chiralities. This new tool takes full advantage of the entire machinery and features of the CRYSTAL package, especially with regard to the use of symmetry, with a great saving of time and computational resources and the consequent ability to completely characterize large systems.

As a first application, we carried out the study of multiwall carbon nanotubes (MWCNT), as a prototype of the material of both scientific and technological interest.

Carbon nanotubes^15,16^ (CNT) have many potential applications in different fields, including biomedical sensor, storage and energy conversion devices, nanoscale molecular sieves, additives for polymeric bracket materials in catalytic processes, etc.^17−19^ Their synthesis is often accompanied or directed to the formation of multiwall structures, MWCNT. These can have some characteristics similar to those of the single wall but with greater structural stability and uniformity. Or they may have a specific peculiarity, such as a lower thermal conductivity,^20,21^ which make them interesting materials from the point of view of technological applications; in this case as thermoelectric materials.^22^ So, in documenting and exploring the limitations and potential of the new tool, we have also provided a first glimpse of challenging problems such as the spectroscopic characterization of MWCNT and the engineering of semiconductor MWCNT to be exploited in thermoelectric devices.

The paper is structured as follows: in the next section, the method to model nanotubes is revised and generalized to the case of multiwall systems. Then, the algorithm is tested on different kinds of MWCNT, which are characterized with regard to their structural, electronic, dynamical, and transport properties. Results are compared with the experimental and/or theoretical data, when available in the literature.

## 2 Methods

### 2.1 Theory

CRYSTAL is a computational tool for solid-state chemistry and physics, based on an original expansion of the crystalline wave function to a set of localized Gaussian-type orbitals, centered on each atom of the unit cell. Hartree–Fock (HF), density functional theory (DFT), and hybrid methods are available at a low computational cost due to full exploitation of point and translational symmetry, both in the direct and reciprocal space. In addition, CRYSTAL can deal, at the same level of accuracy, with different dimensions (D): zero-dimensional (0D) (molecules and polymers) one-dimensional (1D) (nanotubes and nanowires) two-dimensional (2D) (surfaces) and three-dimensional (3D) (bulk). In the case of nanotubes, the use and exploitation of the additional helical symmetry have provided a double benefit: (i) a particularly friendly and simple input and (ii) the possibility to simulate very large tubes.

Nanotubes are cylindrical structures periodic along a single direction, usually defined as *x*. They can be modeled by wrapping the corresponding 2D layer along the rolling vector, **R**, defined as **R** = *n*_1_**a**_1_ + *n*_2_**a**_2_, where **a**_1_ and **a**_2_ are the lattice vectors of the slab unit cell and (*n*_1_, *n*_2_) are integer numbers that fully define the nanotube. In fact, |*R*| is the circumference and the chiral angle, θ, is defined as the angle between **R** and **a**_1_.^23^ According to Hamada et al.,^24^ the chirality can be defined as follows: armchair (*n*_1_, *n*_1_), zigzag (*n*_1_, 0), or chiral (*n*_1_, *n*_2_). So, from **R**, the nanotube diameter *D* = |*R*|/π and the angle θ can be calculated as
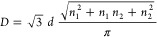
1
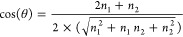
2where *d* is the C–C bond length. Then, depending on **R**, two other lattice vectors are uniquely defined in terms of 4 integers: (i) the nanotube lattice parameter **L**, chosen as the shortest vector perpendicular to **R** and defining the periodicity along *x*: **L** = *l*_1_**a**_**1**_ + *l*_2_**a**_**2**_ (with *l*_1_ and *l*_2_ integers); (ii) the helical (i.e., roto-translational) vector **H** = *h*_1_**a**_**1**_ + *h*_2_**a**_**2**_, which posses a rotational component along the circumference vector, **R**, and a translational component along the lattice parameter, **L**, and then determines the correspondence between a translation in the flat slab with a roto-translation on the curved surface.

The periodicity along the tube axis, i.e., the existence of the longitudinal vector **L**, is not satisfied for all possible 2D (slab) lattices. In fact, the orthogonality condition between **R** and **L** provides the following equation
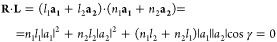
3

However, this equation cannot be satisfied for any combinations of **a**_**1**_, **a**_**2**_, and γ. The equation generates the *l*_1_/*l*_2_ ratio, which is a rational number, whereas cos γ and **a**_**1**_, **a**_**2**_ are real numbers. This is the reason why, among the five 2D Bravais lattices, the hexagonal and square ones are the only ones that can be wrapped in any chirality (*n*_1_, *n*_2_), whereas the rectangular and rhombohedral can only give rise to (*n*_1_, *n*_1_) and (*n*_1_, 0) nanotubes, respectively, and it is not possible to roll up any tube starting from an oblique lattice.^25^

**Figure 1:**
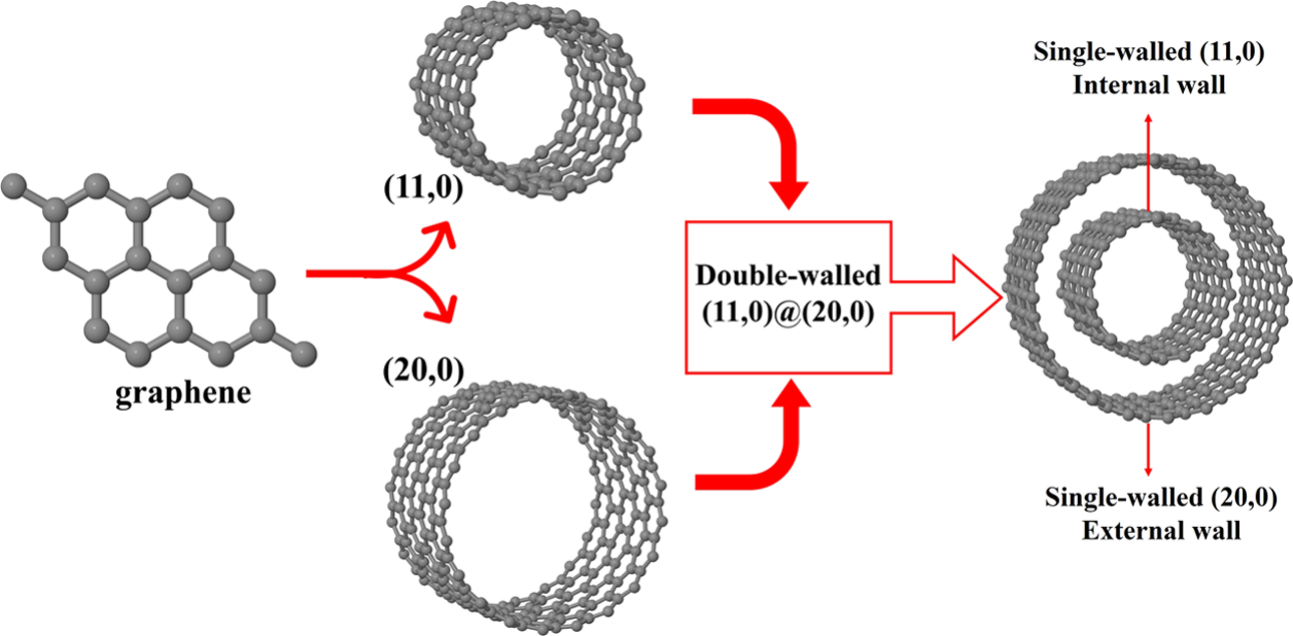
Rolling up a multiwall nanotube structure with CRYSTAL.

Starting from the same theory used for the single-wall nanotubes, the implementation of the multiwall nanotubes follows. The novelty consists of the possibility to generate *M* separately single-wall tubes, starting from a given 2D (or 3D by cutting the proper slab) system, according to the rules just outlined, as shown in Figure 1. That is, once the rolling vector **R** of each wall is defined, the code calculates the corresponding **L** and **H** vectors, and from the atoms in the asymmetric unit generates the full nanotube.

The self-consistent field (SCF) cycle becomes a double-step procedure: first, the wave function of each wall, with its own symmetry, is calculated; then, eventually, using as initial guess the density matrix just calculated for each tube, the energy of the whole multiwall structure is computed. In the last step, only common symmetry operators are kept. For sake of clarity, the corresponding input examples are given in Table S1 of the Supporting Information (SI).

In the case of geometry optimization, two procedures have been implemented. The first, ruled by the OPTWALL keyword, is a full optimization (atomic coordinates, cell parameters, and volume) of each wall, as a separate moiety. Therefore, with this option, each wall is relaxed as if it were isolated. The second, controlled by the keyword OPTMULTI, performs a complete optimization of the entire multiwall system, and only the common symmetry operators (if any) are retained along the process. The two strategies, documented in Table S2, can be used together in the search for the minimum energy configuration, potentially saving a lot of CPU time.

Besides, nanotube walls can be manipulated and reoriented with respect to the others using two new keywords. ROTWALL allows rotating the nanotube wall of any angle between 0 and 360° along the periodic axis, *x*. TRANSWALL performs a rigid shift of the atomic position of the selected wall along the *x*-axis. Both options can be used in association with the optimization keywords to optimize the interlayer stack. Input examples are reported in Table S3.

### 2.2 Computational Setup

The calculations are performed with a β version of the CRYSTAL code, using both the generalized gradient approximated Perdew–Burke–Ernzerh (PBE) functional^26^ and the global hybrid B3LYP,^27^ which includes 20% of the exact exchange. To account for dispersion, the B3LYP-D3 functional, as originally proposed by Grimme^28^ and implemented in the code, is also employed. Carbon atoms are described by the standard all-electron basis set 6-21G*.^29^

The DFT integration is performed within a grid containing 99 radial and 1454 angular points, as specified by the XXLGRID keyword.^30^ The accuracy of the truncation criteria for the bielectronic integrals, Coulomb and HF exchange series, is controlled by a set of five thresholds for which the strict values of [8, 8, 8, 8, 16] are adopted. In the self-consistent field (SCF) procedure, the shrinking factor for both the diagonalization of the Fock matrix and the calculation of the energy is set to 4, corresponding to 4 independent *k*-points in the irreducible part of the Brillouin zone. The total and projected density of states (DOS) and the band structure are plotted using the same *k*-point sampling as in the SCF. The vibrational frequencies at the Γ point were computed within the harmonic approximation by diagonalizing the mass-weighted Hessian matrix.^31,32^ Intensities were evaluated with a Berry phase approach by the calculation of the atomic Born tensors, and the corresponding infrared (IR) spectra are produced using a Lorentzian shape with a full width at half-maximum (FWHM) of 10 cm^–1^ attributed to each peak.^33^

The Seebeck coefficient (*S*) and the power factor (PF) of semiconductor MWCNTs were calculated using the semiclassical Boltzmann transport equation (BTE) theory,^34^ the frozen band approximation, and assuming the energy relaxation time for carriers as a constant parameter, derived on the basis of experimental measurements.^35^

Carbon nanotubes are obtained from the roll-up of a single sheet of graphite, i.e., graphene. Graphite belongs to the hexagonal space group *P*6_3_/*mmc* with unit cell parameters *a* = 2.47 *Å* and *c* = 6.60 *Å*, and consists of flat layers of hexagons of carbon atoms. In each layer, the sp^2^-hybridized carbon atoms are covalently bonded to three other carbon atoms. Starting from the graphite bulk, it is possible to cut a one-layer slab, orthogonal to the *c* axis, characterized by the four Miller indexes [0001], and roll it up to design nanotubes of any chirality. As an alternative, carbon nanotubes can be wrapped starting from the slab, belonging to the point group *P*6/*mm* with cell parameter *a* = 2.47 Å. The input files for both possibilities are reported in Table S1.

It is worth noting that CNT are usually built starting from a hexagonal cell, with γ = 60°, whereas the adopted convention in CRYSTAL is γ = 120°, and this has to be carefully considered while choosing the *n*_1_ and *n*_2_ parameters that define the **R** rolling vector.^2^

Multiwall carbon nanotubes (MWCNT) are used as a test case to verify the reliability of the implemented algorithm and explore the generality of the method. Calculations were performed on a set of double-wall (DW) systems, considering the tubes obtained from both the bulk and the slab and with different chiralities, i.e., armchair, zigzag, and chiral. Then, to investigate the influence of the number of walls on the electronic structure and mechanical properties, starting from the most stable armchair and zigzag double-wall systems, a set of MWs of increasing diameter, with *M* > 2, has been designed and characterized.

Graphene (gr), and the corresponding single-wall(SW) tubes, were simulated and optimized at the same computational level to allow a fruitful and unavoidable comparison. In particular, the multiwall stability is discussed according to the following two quantities. The formation energy per atom, *E*_form_, is defined as the energy difference with respect to an optimized *M*-layers slab of graphene
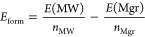
4where *E*(MW) and *E*(Mgr) are the energies of the optimized *M*-wall nanotube and *M*-layer graphene, respectively, and *n*_*x*_ are the number of atoms in the corresponding reference cells. The inter-wall energy, *E*_iw_, which is an estimate of the interaction between walls, normalized on the number of walls, *M*
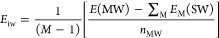
5where *E*_*M*_(SW) are the energies of the isolated single-wall nanotubes. In this case, an estimate of the basis set superposition error (BSSE) is provided adopting the counterpoise method, proposed in the 1970s by Boys and Bernardi.^36,37^ The corrective term, *E*_BSSE_(SW), is calculated for each wall as
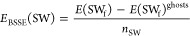
6and added to *E*_iw_. The two quantities in the equation above refer to the energy of the single nanotube frozen in the final MW optimized geometry, isolated, *E*(SW_f_), and surrounded by ghost functions placed in the same position of the other walls, *E*(SW_f_)^ghosts^, respectively.

## 3 Results and Discussion

### 3.1 Internal Check

After several tests on different structures, based on their relative stability, we have selected three double-wall carbon nanotubes, namely (7,7)@(12,12), (12,6)@(20,10), and (11,0)@(20,0) as the representative of different chiralities; see Table 1. The internal consistency and the accuracy of the computational scheme were verified by exploring the following possibilities: (A) tubes are built starting from the 3D or 2D precursor; (B) relaxed or unrelaxed *M*-layers are used as a starting point; (C) the two optimization procedures (OPTWALL+OPTMULTI vs OPTMULTI) are compared.

**Table 1:** Structural Properties of the Armchair, Zigzag, and Chiral Double-Wall Nanotubes^a^

	*n*_AT_	C–C	*C-Ĉ-C*	*d*_IW_	*D*_in_	*D*_ext_	*E*_gap_	*E*_form_	*n*_sym_	*n*_cyc_	CPU
Armchair											
(6,6)@(12,12)	72	1.43	119.6	4.08	8.28	16.44	0.0	0.062	24	7	806
(7,7)@(12,12)	76	1.43	119.7	3.46	9.69	16.48	0.0	0.056	4	10	5804
(8,8)@(12,12)	80	1.44	120.3	3.02	10.79	16.66	0.0	0.076	16	11	2490
(9,9)@(12,12)	84	1.44	119.7	2.06	11.79	17.35	0.0	0.162	12	14	5626
Zigzag											
(10,0)@(20,0)	120	1.43	119.9	3.93	7.95	15.81	0.45	0.080	40	12	2650
(11,0)@(20,0)	124	1.43	120.0	3.63	8.76	15.84	0.34	0.076	4	13	10 494
(12,0)@(20,0)	128	1.43	120.1	3.26	9.47	15.84	0.07	0.085	32	33	6281
(13,0)@(20,0)	132	1.45	119.1	3.03	10.16	16.09	0.02	0.095	4	15	23 277
(15,0)@(20,0)	140	1.47	121.5	2.81	11.37	16.78	0.00	0.200	20	25	11 345
Chiral											
(6,12)@(10,20)	448	1.43	119.7	4.16	12.57	20.89	0.11	0.043	8	11	450 176
(7,14)@(10,20)	476	1.42	121.0	3.12	14.56	20.80	0.16	0.050	2	13	1 460 165

a The number of atoms (*n*_AT_), bond lengths (C–C), bond angles (*C-Ĉ-C*), inter-wall distances (*d*_IW_), internal and external diameters (*D*_in_ and *D*_ext_), the fundamental energy gap, *E*_form_, at the PBE level, and the number of symmetry operators (*n*_sym_) are reported. Distances are in Å, angles in degrees, and energies in eV. For a single SCF, the number of cycles, *n*_cyc_, and the total CPU time, in seconds, on 16 processors of an Intel Xeon 3.00 GHz cluster, are reported.

The three strategies provide the same final results in terms of total energy, atomic positions, lattice parameters, band gap (*E*_gap_), and Mulliken charges. The difference is in the required CPU time, which for the present simple models is not essential but can become crucial when hundreds or thousands of atoms are involved. It is worth saying that the very high symmetry characterizing each tube, which is fully exploited during the OPTWALL procedure, is generally lost in the MW calculation in which few (if any) symmetry operators are retained.

With regard to (C) tests, the two optimization procedures provide equivalent results, as documented in Figure S1, and the OPTWALL + OPTMULTI option is the most time-efficient.

Finally, the TRANSWALL and ROTWALL options, which can become a useful tool for materials with more complex structures that may require elaborate modeling, were tested. In the case of carbon wall nanotubes, the possibility of exploring these further degrees of freedom is particularly interesting because of the well-known influence of the interlayer stacking on the main properties of graphene-like materials.^38,39^

**Figure 2:**
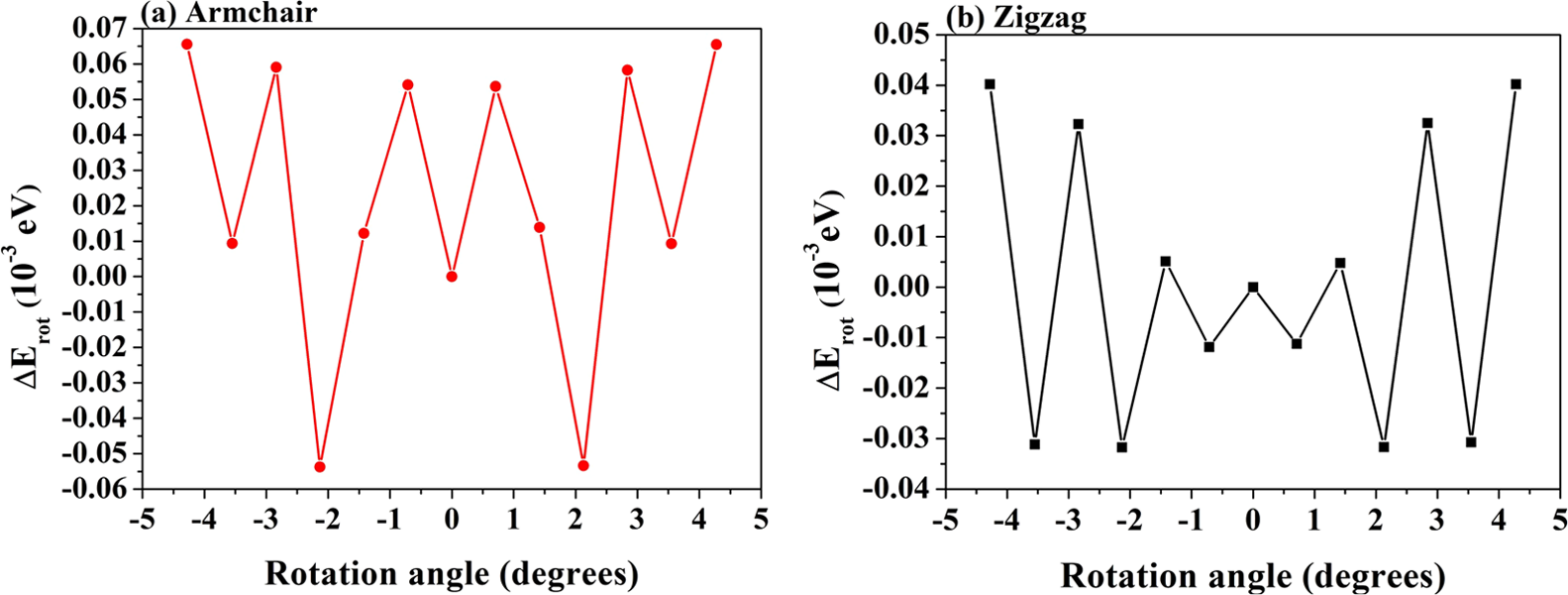
Energy dependence (PBE, in meV) on the orientation of the outer tube. The same trend is calculated at the B3LYP-D3 level. Left and right panels represent the armchair (7,7)@(12,12) and zigzag (11,0)@(20,0) DW nanotubes when the outer wall is rotated between −4.3° < γ < 4.3° while the inner wall is kept fixed.

The translation along the periodic axis occurs without any energy barrier, both at the PBE and B3LYP-D3 levels. Instead, by rotating the outer wall of the armchair (7,7)@(12,12) and of the zigzag (11,0)@(20,0) nanotubes between −4.3 and 4.3°, we obtained the energy curves as in Figure 2. This angle represents the difference in degrees between two equivalent inner/outer-wall configurations. In the case of armchair (zigzag), a rotation of 2° yields a configuration that is 0.05 (0.03) eV more stable than the starting one, indicating that a favorable stacking of the layers can significantly stabilize the structure.

### 3.2 Structures and Energies

The first set of calculations was performed on double-wall (DW) nanotubes exploring the three chiralities, armchair, zigzag and chiral, and the effect of the inter-wall distance. The main results are collected in Table 1 and Table S4.

In general, the distortion with respect to graphene is uniformly distributed over all of the atoms of the lattice as imposed by symmetry. Bond angles and distances deviate by only a small amount from those of the planar geometry. In particular, the distances between adjacent carbon atoms are in the range of 1.42–1.47 Å, while in the two layers of graphite, the average C–C length is 1.43 Å. The *C-Ĉ-C* angles present a small deviation from the ideal value of 120°.

The effect of symmetry on the CPU time is evident when comparing the (6,6)@(12,12) and (7,7)@(12,12) DW nanotubes, which have 24 and 6 symmetry operators, respectively. Given the same number of SCF cycles to converge and a negligible difference in the number of atoms in the cell, it can be argued that the CPU speed-up is proportional to the ratio of the number of symmetry operators.

The formation energy of these systems is found as a balance between two contributions, the strain energy, defined as the force to wrap *M*-flat surfaces, and the inter-wall interaction. Regardless of the functional adopted, *E*_form_ is always positive indicating that a given amount of energy is needed to synthesize these materials starting from their 2D precursor. As expected, the less stable structures, with higher *E*_form_, are those with the smallest inter-wall distance and the most distorted geometries in terms of bond lengths and angles.

Interestingly, the two structures with the lowest formation energy, the armchair (7,7)@(12,12) and the zigzag (11,0)@(20,0), have an interlayer distance of *d*_iw_ = 3.46 and 3.63 Å, respectively. These values are extremely close to that of 3.35 Å measured in graphite^40^ and of 3.48 Å recently determined for bilayer graphene^41^ and only slighter longer than the experimental inter-wall distances of 3.41–3.35 Å observed for multiwall carbon nanotubes by Saito et al.^42^ The good agreement with the experimental data confirms that the algorithm is able to predict the most likely structures. It can therefore be concluded that (7,7)@(12,12) and (11,0)@(20,0) double-wall systems are almost iso-energetic, so that both armchair and zigzag chiralities can be obtained, as already stated in the previous work on single-wall carbon nanotube^9^ and confirmed by several experimental findings.^43^

**Figure 3:**
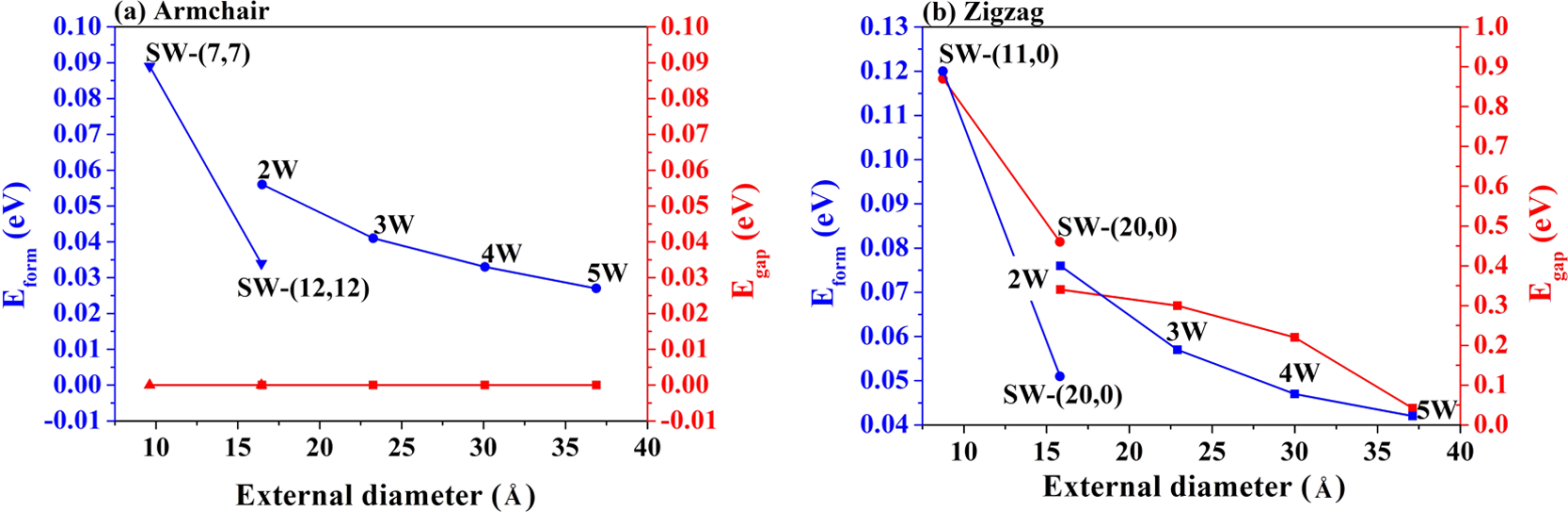
*E*_form_, at the PBE level, for armchair and zigzag MW nanotubes of an increasing number of walls. For both chiralities, *E*_gap_ as a function of the outer wall diameter is also reported (red curves).

Then, starting from (7,7)@(12,12) and (11,0)@(20,0), two sets of nanotubes up to 5 walls, of increasing outer diameter, are drawn, keeping the distance between the walls equal to the optimal value of *d*_iw_ = 3.46 and 3.63 Å for armchair and zigzag, respectively. Structural details are reported in Table S5. The largest 5*W* tubes have a diameter of ≈37 Å, already in the order of some synthesized experimental samples.^44,45^ The formation energies, computed according to eq 4, using a fully optimized *M*-layer sheet of graphite as a reference system, are shown as a function of the MW diameter in Figure 3. In accordance with experimental observation, *E*_form_ decreases as the number of walls increases. For the largest armchair and zigzag systems, containing 340 and 580 atoms in the reference cell, respectively, *E*_form_ = 0.03 and 0.04 eV/per atom.

Finally, to explore the inter-wall interaction as a function of the inter-wall distance, a set of armchair (7,7)@(X,X) characterized by outer walls at an increasing distance was designed, ranging from *d*_iw_ = 2.85 (for *X* = 11) to *d*_iw_ = 15.8 (for *X* = 30). Structural and energetic details of this DW family, as evaluated at the PBE, B3LYP, and B3LYP-D3 levels, are reported in Table 2. For all functionals, the BSSE correction does not change either the general trend or the position of the minimum, resulting in negligible inter-wall distances up to 5 Å. The interlayer distances, as evaluated at the B3LYP-D3 level, are slightly shorter than those in PBE and B3LYP geometries. On the contrary, the functional adopted is crucial in determining the correct energy balance of these structures. As expected, an attractive interlayer interaction is calculated only at the B3LYP-D3 level, confirming the fundamental role played by the dispersive forces in these materials.

**Table 2:** The (7,7) Tube Contains 28 Carbon Atoms. Number of Atoms, nAT, Inter-wall Distance, dIW, Eform and Inter-wall Energy BSSE Corrected as Evaluated at the PBE, B3LYP and B3LYP-D3 Level Are Reported. Energies Are in eV per Atom

		PBE			B3LYP			B3LYP-D3		
	*n*_AT_	*d*_IW_	*E*_form_	*E*_iw_^BSSE^	*d*_IW_	*E*_form_	*E*_iw_^BSSE^	*d*_IW_	*E*_form_	*E*_iw_^BSSE^
@(11,11)	72	3.01	0.089	0.0568	2.96	0.080	0.0747	2.89	0.056	–0.0119
@(12,12)	76	3.41	0.056	0.0155	3.51	0.034	0.0203	3.40	0.030	–0.0221
@(13,13)	80	4.11	0.050	0.0024	4.07	0.031	0.0119	3.38	0.039	–0.0193
@(14,14)	84	4.76	0.049	0.0001	4.94	0.021	0.0007	4.74	0.049	–0.0109
@(15,15)	88	5.63	0.046	0.0002	5.44	0.018	0.0003	5.47	0.051	–0.0059
@(16,16)	92	6.13	0.044	0.0002	6.13	0.015	0.0002	6.16	0.051	–0.0035
@(18,18)	100	7.51	0.040	0.0001	7.49	0.010	0.0001	7.48	0.049	–0.0014
@(20,20)	108	8.85	0.036	0.0000	8.93	0.007	0.0001	8.83	0.046	–0.0006
@(30,30)	148	15.83	0.026	0.0000	15.91	0.001	0.0000	15.65	0.003	0.0000

### 3.3 One-Electron Properties

**Figure 4:**
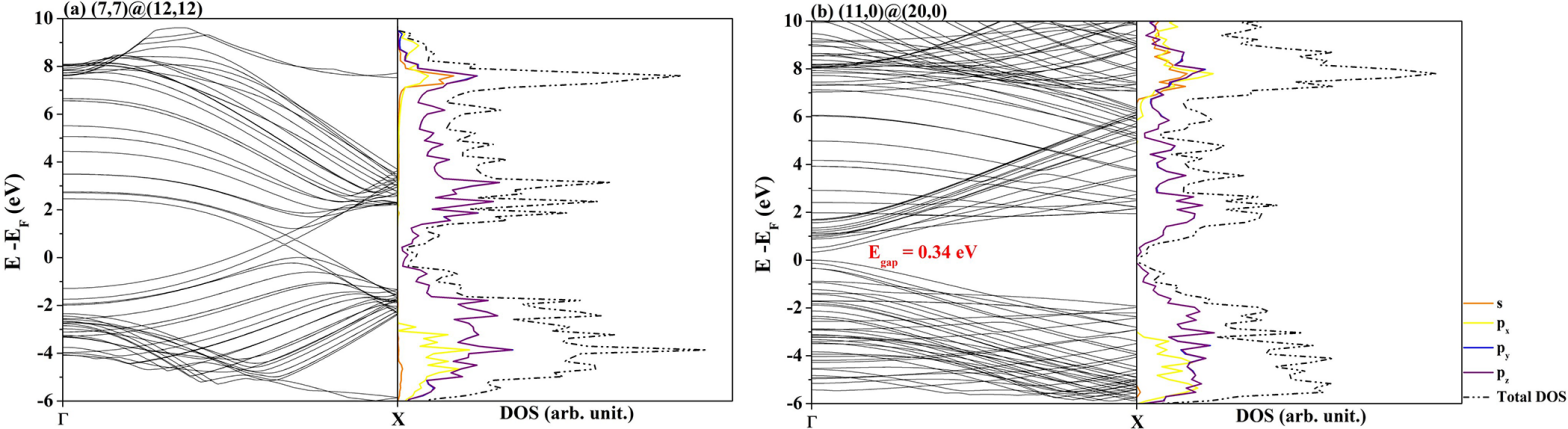
Band structure and density of states for armchair (7,7)@(12,12) (left) and zigzag (11,0)@(20,0) (right) DW nanotubes. The corresponding features for the single-wall tubes are reported in Figure S3. To facilitate comparison, the bands have been shifted to bring the Fermi level of each system to coincide with zero.

The band structure and density of states (DOS) of the most stable DW systems, namely the (7,7)@(12,12) and (11,0)@(20,0), are calculated and discussed with reference to the corresponding properties of the single-wall nanotubes. The results are summarized in Figure 4.

In the case of armchair SW nanotubes, there are two evident Dirac cones, one in the conduction band and the other in the valence band, that intersect each other at a single *k* point, determining the metallic behavior of these systems; see Figure S3. In the DW structure, the Dirac cones of the two nanotubes almost overlap, giving rise to two points of intersection and an enhancement of the conductivity.

Zigzag SWs present a band gap that decreases as the diameter of the tube increases. The resulting DW structure retains the semiconductor character, although the *E*_gap_ is smaller than those of the two isolated tubes.

The projected density of states (PDOS) on the different atomic orbitals of the carbon atoms can provide useful information on the band composition around the Fermi surface. The p_*y*_ and p_*z*_ are symmetry-equivalent and their contributions are equal, whereas p_*x*_ is oriented along the periodic direction. Also in the case of PDOS, both the armchair and zigzag DWs conserve the main features of the SW constituents. In particular, *s* shells contribute to the lowest part of the valence band, p_*x*_ orbitals contribute to the region slightly higher in energy, and p_*y*_ and p_*z*_ are the ones responsible for the bands immediately below and above the Fermi level. These last bands determine most of the properties related to electronic mobility (chemical reactivity but also conductivity) and are indeed particularly interesting.

In the case of zigzag MWCNT, the band gap decreases as the number of walls increases, ranging from 0.30 eV for *M* = 3 to 0.08 eV for *M* = 5; see Table S5. The persistence of a semiconductor character in these rather stable nanostructures paves the way for the study of their transport properties and potential thermoelectric performance.

### 3.4 Lattice Dynamics

IR and Raman frequencies in Γ were calculated at the PBE level for two sets of nanotubes: the armchair [(7,7) and (12,12) SW, (7,7)@(12,12) and (7,7)@(12,12)@(17,17) MW] and the zigzag [(11,0) and (20,0) SW, (11,0)@(20,0) and (11,0)@(20,0)@(29,0) MW].

The first-order experimental Raman spectrum of carbon nanotubes exhibits a line at 1582 cm^–1^ due to the stretching of the C–C bond.^46^ Our calculated values, around 1520 and 1540 cm^–1^ for armchair and zigzag, respectively, are in rather good agreement with the experimental findings; see Figure S5. The weak interactions between layers have almost no effect on the MW Raman fingerprint, as can be seen from the coincidence between the DW and the single-wall signals. Furthermore, the difference between the two sets is below the numerical precision, and therefore, it is practically impossible to determine the chirality by calculating the Raman spectra of such materials.

**Figure 5:**
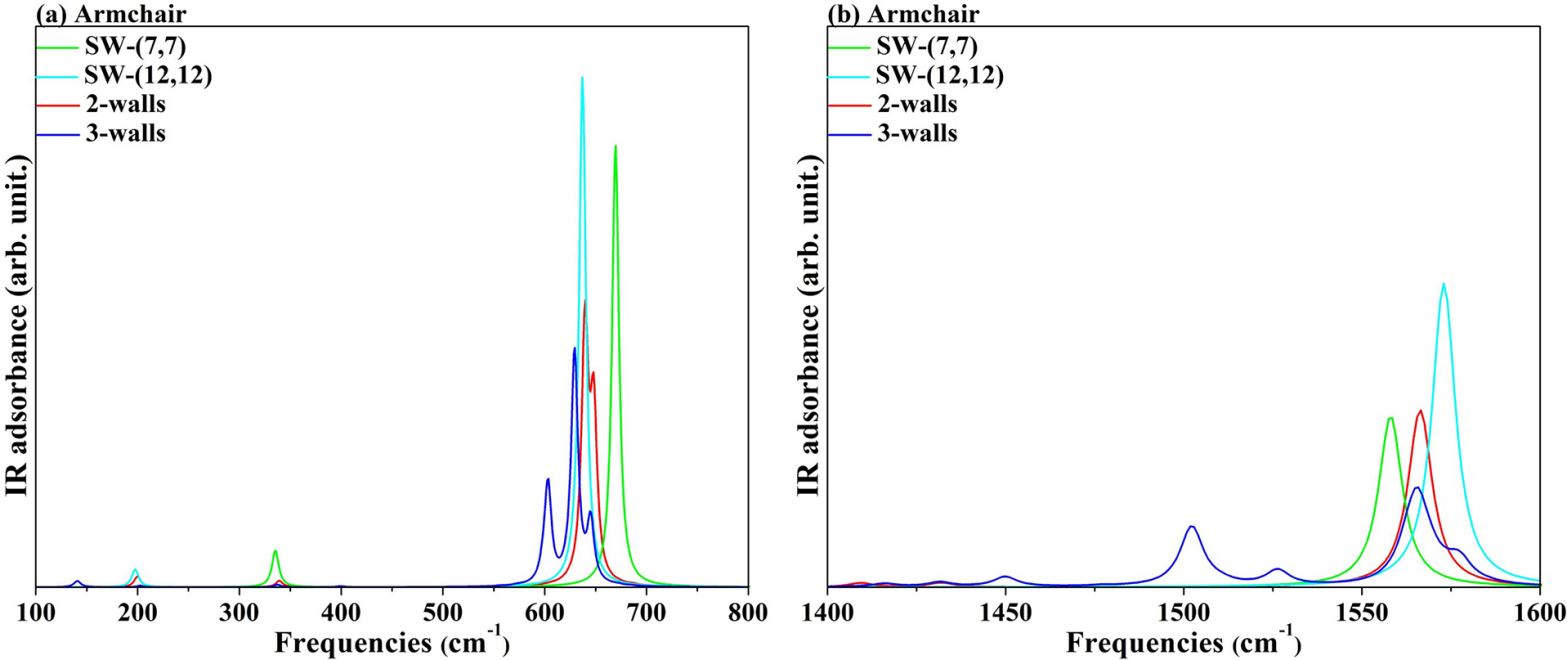
IR spectra of 2*W* and 3*W* nanotubes, at the PBE level, are shown in the region of the soft modes (left panel) and high frequencies (right panel). The spectra of the single-wall (7,7) and (12,12) are added for comparison.

The IR spectra for armchair and zigzag, shown in Figures 5 and S4, are qualitatively in good agreement with the experimental vibrational modes obtained by Kastner et al.^46^ for carbon nanotubes. Transmission infrared spectra show one broad and asymmetric line at 1575 cm^–1^ and a line at 868 cm^–1^. Interestingly, we found IR signals at 1550 and 680 cm^–1^ only for the armchair nanotubes, while th*e* zigzag spectra showed a single intense peak around 690 cm^–1^. This substantially different spectral profile, whose shape is maintained as the number of walls increases, could allow the identification of the chirality of the nanotube without ambiguity.

Moreover, despite the low values of *E*_iw_, the spectral fingerprint of 2*W* and 3*W* tubes presents original features that could help determine the thickness of the nanostructures. In the armchair tubes, the signal corresponding to the wagging out-of-plane vibration of the carbon atoms (680 cm^–1^ for SW) becomes a doublet, and then a triplet, for the 2*W* and 3*W* multiwall. In addition, in the region of the soft modes, two/three small but distinct peaks emerge at their respective single-wall signals. For zigzag structures, new down-shifted signal(s) appear, corresponding to the same displacement of the carbon atoms, as occurring in the 2(3) tubes.

To summarize, IR spectroscopy can provide clear information on both the chirality and thickness of MW nanotubes.

### 3.5 Transport Properties

**Figure 6:**
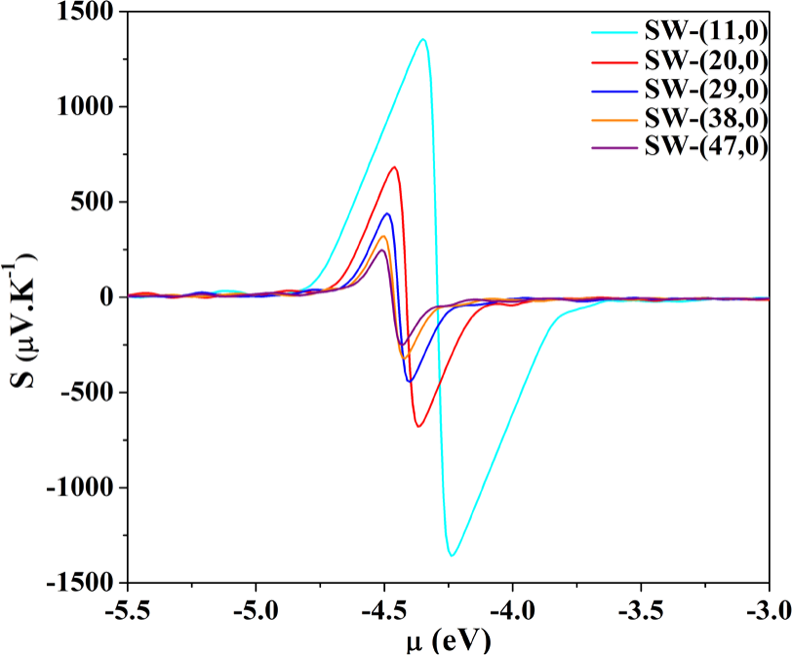
Seebeck coefficient at 300 K for zigzag SW nanotubes of increasing diameter. The peak value for (11,0) *S* = 1300 μV K^–1^, which is in perfect agreement with that calculated by Hung et al. for the same system.^50^

We explored the thermoelectric performance of semiconducting MWCNT in the frame of the Boltzmann transport formalism.^47^ To validate our method and evaluate the effect of multiple walls on these properties, we first calculated the Seebeck coefficients for SWCNTs. The results, shown in Figure 6, can be summarized as follows: (i) *S* increases with decreasing the diameter of the nanotube as a consequence of the narrowing of the band gap, in qualitative agreement with the recent experimental results;^48^ (ii) in accordance with theoretical predictions,^49,50^ the peak of *S* is nearly an order of magnitude higher than those observed experimentally.^51,52^ To comment on this discrepancy, it can be noted that the Seebeck coefficient is highly sensitive to the position of the Fermi energy, which in turn is controlled by the carrier density. A small shift toward lower values of the chemical potential provides values for the thermopower in the range of the experimental findings. In particular, Nakai et al.^48^ found a positive sign for *S*, indicating hole-like carriers and a value of ≈170 μV K^–1^ for SWCNT with a mean diameter of ≈ 20 Å. Our (29,0) tube has a diameter of 23 Å, and the calculated thermopower at 300 K is *S* ≈ 280 μV K^–1^ for a chemical potential that is 50 meV lower than that of the Fermi level.

**Figure 7:**
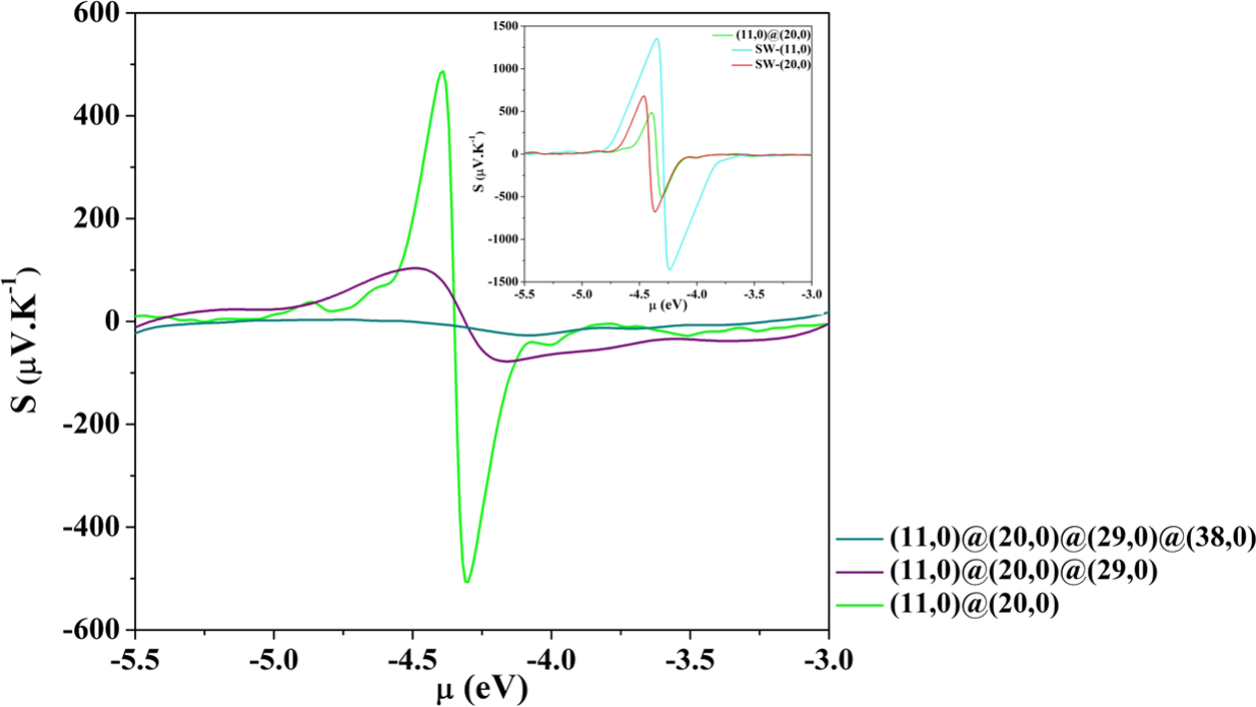
Seebeck coefficient at 300 K for zigzag MW nanotubes (11,0)@(20,0), (11,0)@(20,0)@(29,0), and (11,0)@(20,0)@(29,0)@(38,0). Inset: *S* of (11,0)@(20,0) is compared with those of the two constituent tubes to highlight the different orders of magnitude.

**Figure 8:**
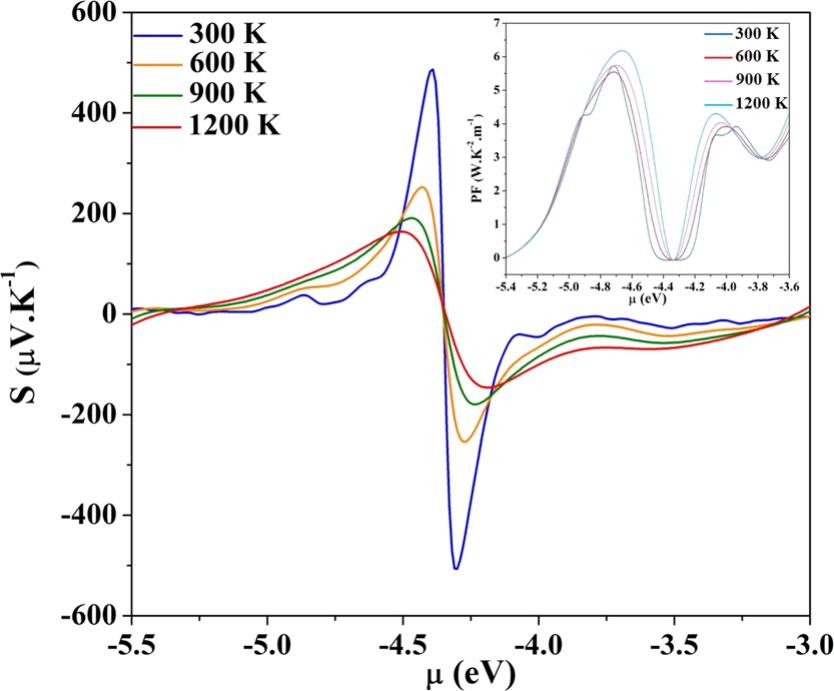
Seebeck coefficient of (11,0)@(20,0) at different temperatures. Inset: the power factor at different temperatures: its peak occurs at −4.7 eV, corresponding to a carrier concentration of 4 × 10^22^ cm^–3^.

Then, we compute the Seebeck coefficient for MWCNTs. As measured in several samples, we found that the thermopower is almost one order of magnitude lower than in SW. Moreover, as the number of walls increases, the progressive narrowing of the gap causes a decrease in *S* with the consequent disappearance of any thermoelectric power. Both these features are documented in Figure 7, where *S* is reported for the zigzag multiwall, with *M* > 4, at 300 K. In the inset, the thermopower of the double wall is compared with that of the SW. The most interesting system seems to be the double-wall (11,0)@(20,0). Its thermoelectric behavior shows interesting features that partially reproduce the experimental results of Miao et al.^22^ The dependence of *S* and of the power factor on temperature is well reproduced and is shown in Figure 8: as T increases, the Seebeck coefficient decreases while the PF increases slightly. Moreover, the value of *S*, corresponding to the maximum of PF, which occurs for a chemical potential of −4.7 eV, is 36.7 μV K^–1^, a value that is within the experimental range of 40–30 μV K^–1^ as reported by Miao.^22^

Based on these preliminary results, interesting insights could be hypothesized that unfortunately fall outside the scope of this paper. In particular, to investigate the effect of different chirality on the band gap and simulate the doping process, which can significantly reduce the thermal conductivity with a sensitive improvement in the thermoelectric figure of merit.

## 4 Conclusions

In this paper, a general-purpose robust scheme to model multiwall nanotubes by rolling up layers cutting from hexagonal, square, and rectangular lattices is presented. The entire set of the CRYSTAL^3^ features can be conveniently used to characterize these one-dimensional periodic materials and to explore their potential technological applications. The full exploitation of helical symmetry allows for a particular user-friendly input design, conveniently reduces the computational cost, and permits the treatment of large systems. In addition, there is always the possibility to perform a consistent internal check of every computed property with respect to its values in the precursor two-dimensional materials.

The algorithm is applied for the first time to a family of systems of technological and scientific interest as the multiwall carbon nanotubes.

This preliminary investigation has shown that by working on chirality, inter-wall distance, and thickness (i.e., number of walls), it is possible to design rather stable semiconductors to be used as innovative materials for promising application in the field of carbon-nanotube-based thermoelectric devices.

## Supporting Information Available

Input examples; a flowchart of the MULTIWALL option, band structure and density of state; IR and Raman spectra of single and multiwall nanotubes; and structural information on the multiwall systems (ZIP)
