# Supplementary material for: Ab Initio Modeling of MultiWall: A General Algorithm First Applied to Carbon Nanotubes
Source: J Phys Chem A. 2021 Apr 28;125(18):4003–12. doi: 10.1021/acs.jpca.1c01682 (PMC8279650; doi:10.1021/acs.jpca.1c01682)
Supplement: Supplementary file 1 — jp1c01682_si_001.zip [file jp1c01682_si_001.zip › SI/jp1c01682_SI.pdf]

Ab Initio Modeling of Multi-Wall: a General Algorithm First Applied to Carbon Nanotubes

Naiara Leticia Marana[a,c], Yves Noel[b], Julio Ricardo Sambrano[a], Chiara Ribaldone[c], Silvia Casassa[*,c]

[a] *Modeling and Molecular Simulation Group - CDMF, São Paulo State University, UNESP, Bauru, SP, Brazil*
[b] *Institut des Sciences de la Terre Paris (iSTeP), Sorbonne Université, Paris, France*
[c] *Theoretical Group of Chemistry, Chemistry Department I.F.M., Torino University, Torino 10124, Italy*

Key words: multiwalled nanotube, DFT, CNT

---

1. Input Examples

Input examples to roll up multi-wall nanotubes are reported in Table S1. The input files for the two optimization strategies are shown in Table S2. Example of multi-wall nanotubes manipulation by means of the *ROTWALL* and *TRANSWALL* keywords are given in Table S3.
For the sake of clarity, the flowchart of the new *MULTIWALL* algorithm is reported in Fig. S1

2. Energies

The energy gain calculated with respect to the unrelaxed structure, ΔE_{opt}, is reported in Fig. S2 for the three chiralities. The two different computational strategies, i.e. *OPTMULTI* and *OPTWALL+OPTMULTI*, yield the same results, in terms of final structure and energy.

The formation energies, E_{form}, and inter-wall interactions, E_{iw}, for the set of *armchair* and *zigzag DW* nanotubes analyzed in the article are reported in Table S4. The counterpoise correction, calculated to account for the basis

*Corresponding author
Email address: silvia.casassa@unito.it (Silvia Casassa)

Model multi-wall nanotubes	
CRYSTAL	SLAB
0 0 0	
194	77
2.46 6.70	2.46
2	1
6 0.0 0.0 0.25	6 0.33333 0.666667 0.0
6 0.33333 0.666667 0.0	**NANOMULTI**
SLAB	2
77	WALL
2.47	1
1	ROLLINGV
6 0.33333 0.666667 0.0	6 6
**NANOMULTI**	WALL
2	2
WALL	ROLLINGV
1	12 12
ROLLINGV	**NANOJMOL**
6 6	**ENDWALL**
WALL	
2	
ROLLINGV	
12 12	
**NANOJMOL**	
**ENDWALL**	

Table S1: CRYSTAL input examples for the carbon nanotube, starting from a 3D (bulk, in the left) or 2D (slab, in the right) structure. (6,6) and (12,12) are the internal and the external walls respectively, forming the multiwalled (6,6)@(12,12) nanotube.

Optimize multi-wall nanotubes		
SLAB	SLAB	SLAB
77	77	77
2.46	2.46	2.46
1	1	1
6 0.33333 0.666667 0.0	6 0.33333 0.666667 0.0	6 0.33333 0.666667 0.0
**NANOMULTI**	**NANOMULTI**	**NANOMULTI**
2	2	2
WALL	WALL	WALL
1	1	1
ROLLINGV	ROLLINGV	ROLLINGV
6 6	6 6	6 6
**OPTWALL**	WALL	**OPTWALL**
WALL	2	WALL
2	ROLLINGV	2
ROLLINGV	12 12	ROLLINGV
12 12	**OPTMULTI**	12 12
**OPTWALL**	**ENDWALL**	**OPTWALL**
**ENDWALL**		**OPTMULTI**
		**ENDWALL**

Table S2: CRYSTAL input examples for different optimization strategies for the (6,6)@(12,12) carbon multi-walls nanotube.

Manipulate multi-wall nanotubes	
SLAB	SLAB
77	77
2.46	2.46
1	1
6 0.33333 0.666667 0.0	6 0.33333 0.666667 0.0
**NANOMULTI**	**NANOMULTI**
2	2
WALL	WALL
1	1
ROLLINGV	ROLLINGV
6 6	6 6
**ROTWALL**	**TRANSWALL**
60.0	1.0
WALL	WALL
2	2
ROLLINGV	ROLLINGV
12 12	12 12
**ENDWALL**	**ENDWALL**

Table S3: CRYSTAL input examples for the manipulation of the nanotubes geometrical configuration in the case of the (6,6)@(12,12) multi-walls.

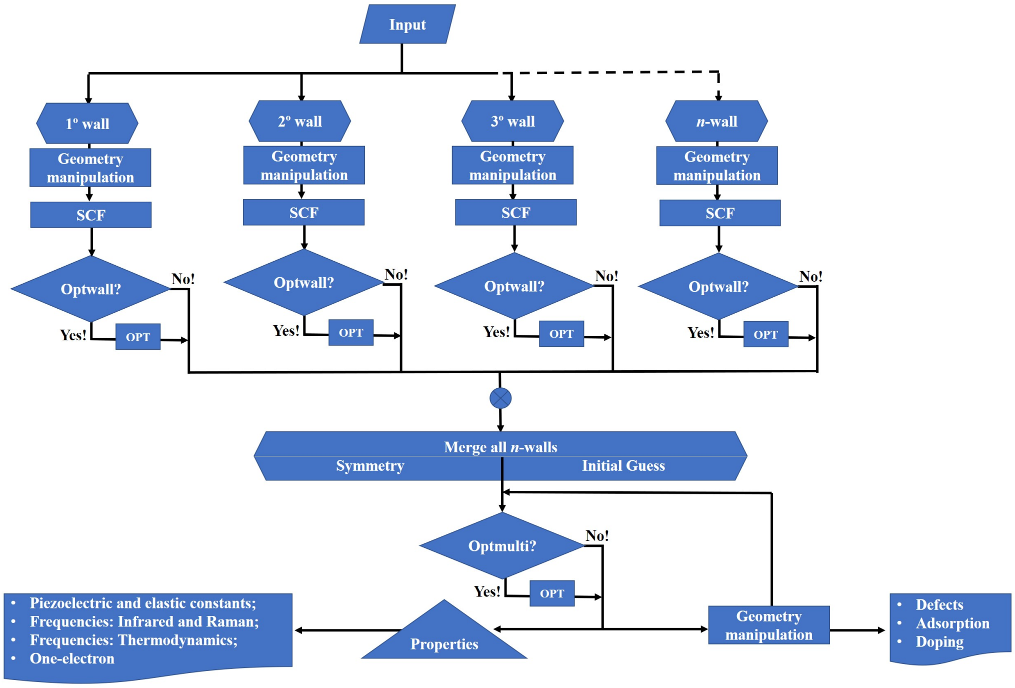

Figure S1: Flowchart of the *MULTIWALL* option in CRYSTAL.

set superposition error, is also reported for sake of comparison.

3. One electron properties

The band structure and density of states of the *armchair* (7,7) and (12,12) and of the *zigzag* (11,0) and (20,0) nanotubes are reported in Fig. S3.

4. IR and Raman spectra

The IR spectra of the*zigzag* multi-wall (11,0)@(20,0) and (11,0)@(20,0)@(29,0) evaluated at the PBE level are shown in Fig. S4. The Raman spectra of the *armchair* (7,7)@(12,12) and *zigzag* (11,0)@(20,0) double-wall nanotubes, evaluated at the PBE level, are reported in Fig. S5. Comparison with the *SW* tubes shows that Raman is poorly sensitive to the thickness of the structure.

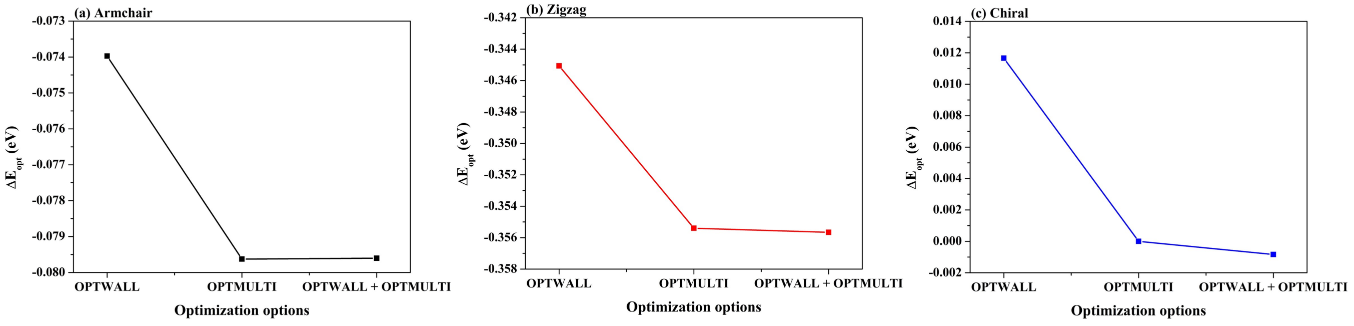

Figure S2: Energy gain, in eV, with respect to the unrelaxed geometries for the *DW* (7,7)@(12,12), (11,0)@(20,0) and (12,6)@(20,10) nanotubes, respectively. The *OPTMULTI* and *OPTWALL+OPTMULTI* strategies provide the same results.

5. Multi wall

The main structural parameters and energetic data of the $MWCNT$, as evaluated at the PBE level, are reported in Tab. S5. All the geometries were fully relaxed.
A summary of the CPU computational time for a complete optimization of each $MWCNT$ is given in Tab. S6. The calculations were performed on 40 CPUs Intel Xeon 2.13 GHz. It is worth to note that the optimization of the single-wall nanotubes is rather straightforward due to the full exploitation of the helical symmetry.

	E_{form}	E_{iw}	BSSE	$E_{\text{iw}}^{\text{BSSE}}$
armchair				
(6,6)(12,12)	0.062	-0.0025	0.0052	0.0027
(7,7)(12,12)	0.056	0.0002	0.0153	0.0155
(8,8)(12,12)	0.076	0.0263	0.0266	0.0529
(9,9)(12,12)	0.162	0.1167	0.0399	0.1566
zig-zag				
(10,0)(20,0)	0.080	-0.0029	0.0075	0.0046
(11,0)(20,0)	0.076	-0.0012	0.0140	0.0128
(12,0)(20,0)	0.085	0.0127	0.0189	0.0317
(13,0)(20,0)	0.095	0.0261	0.0267	0.0527
(15,0)(20,0)	0.200	0.1365	0.0434	0.1799

Table S4: Energies (eV) per carbon atom calculated according to Eq. 4, 5 and 6 of the manuscript, at the PBE level.

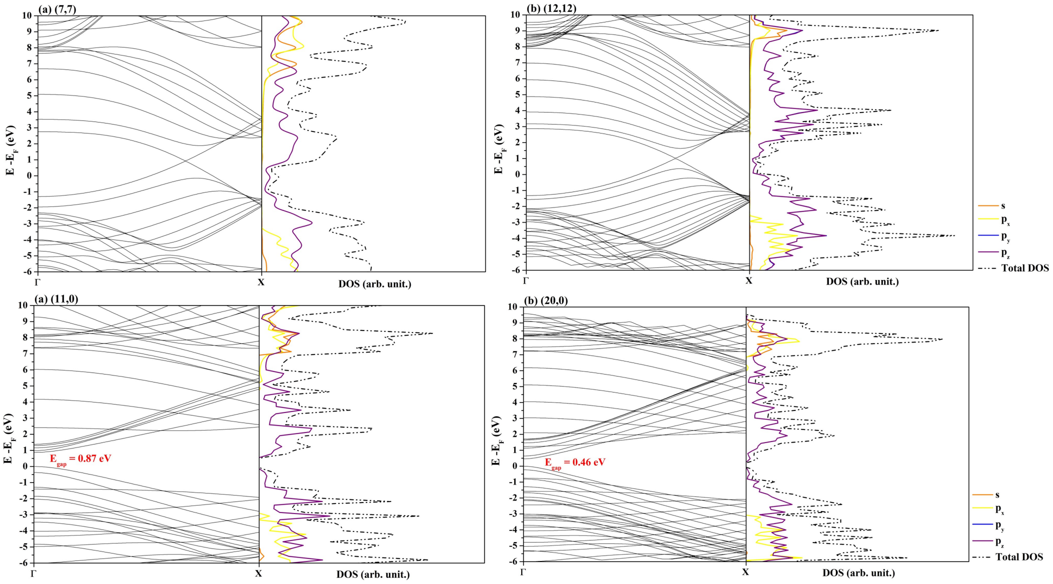

Figure S3: Band structure and density of states of the single-wall carbon nanotubes.

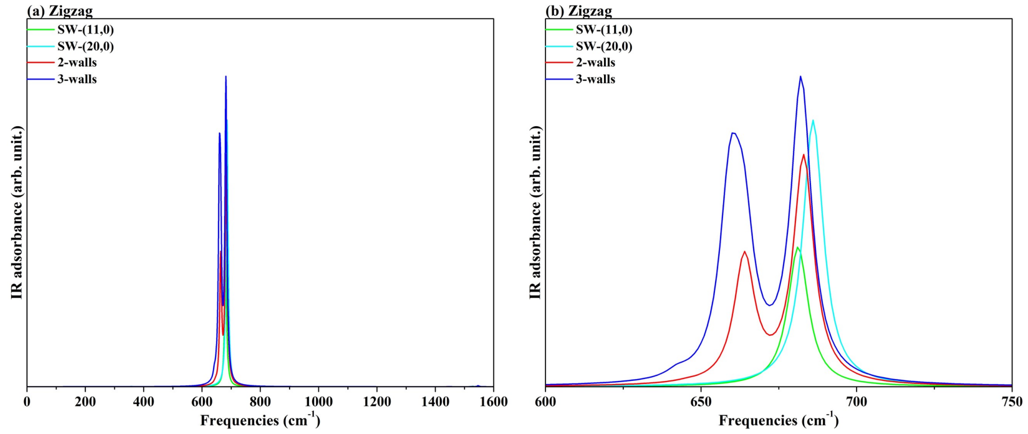

Figure S4: IR spectra at the PBE level of *zigzag* 2W and 3W nanotubes. The fingerprint of the single-wall (11,0) and (20,0) are added for comparison.

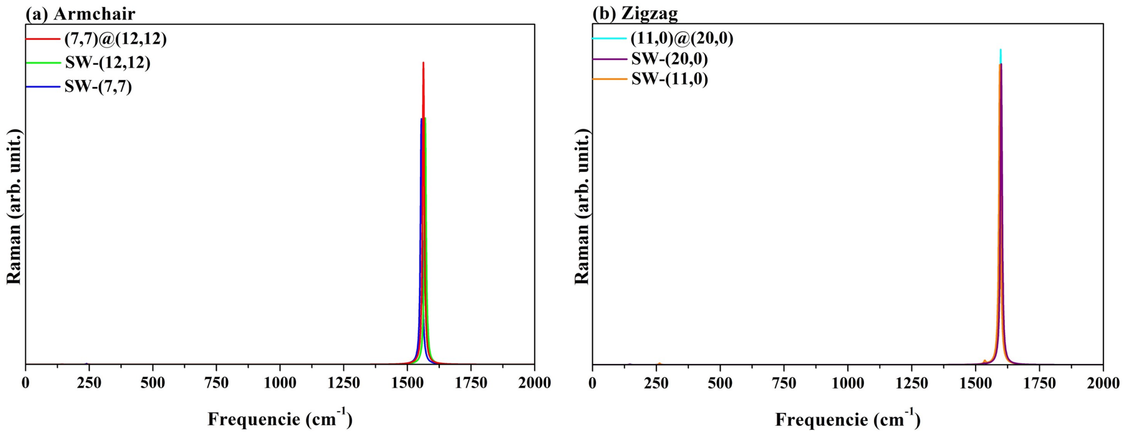

Figure S5: Raman spectra at the PBE level of (7,7)@(12,12) (left) and (11,0)@(20,0) (right) double-walls.

	n_{AT}	D_{ext} (Å)	E_{gap} (eV)	E_{form} (eV)
armchair				
(7)	28	9.62	0.0	0.089
(12)	48	16.44	0.0	0.034
(7)(12)	76	16.48	0.0	0.056
(7)(12)(17)	144	23.26	0.0	0.041
(7)(12)(17)(22)	232	30.08	0.0	0.033
(7)(12)(17)(22)(27)	340	36.88	0.0	0.027
zig-zag				
(11)	44	8.74	0.87	0.120
(20)	80	15.81	0.46	0.051
(11)(20)	124	15.84	0.34	0.076
(11)(20)(29)	240	22.91	0.30	0.057
(11)(20)(29)(38)	392	30.00	0.22	0.047
(11)(20)(29)(38)(47)	580	37.12	0.08	0.042

Table S5: Number of atoms in the *MW* reference cell, outer diameter, D_{ext} (in Å) energy gap and formation energy (eV) as evaluated at the PBE level are reported. To named each *MW*s a simplified nomenclature is adopted. The @ symbol is skipped, for *armchair* only $n_1(=n_2)$ is reported and for *zig-zag* the zero is omitted.

zig-zag	n_{AT}	n_{sym}	*OPTW*	*OPTM*
(11)	44	88	15	–
(11)(20)	124	160	50	2000
(11)(20)(29)	240	232	60	10400
(11)(20)(29)(38)	392	304	90	225209
(11)(20)(29)(38)(47)	580	376	122	1409869

Table S6: PBE parallel calculation on 40 CPU Intel Xeon 2.13 GHz. Number of atoms in the reference cell of the *MW*, n_{AT} and number of symmetry operators, n_{sym}, of the outer wall. The number of symmetry operators reduces to 4 in the case of *MW* systems. In each row, it is given the time per CPU (in sec) for the outer wall relaxation (*OPTW*) and for the optimization of the entire *MW* (*OPTM*). For the adopted nomenclature, see Table S5.
