# Supplementary figures and images for: Ab Initio Modeling of MultiWall: A General Algorithm First Applied to Carbon Nanotubes
Source: J Phys Chem A. 2021 Apr 28;125(18):4003–12. doi: 10.1021/acs.jpca.1c01682 (PMC8279650; doi:10.1021/acs.jpca.1c01682)

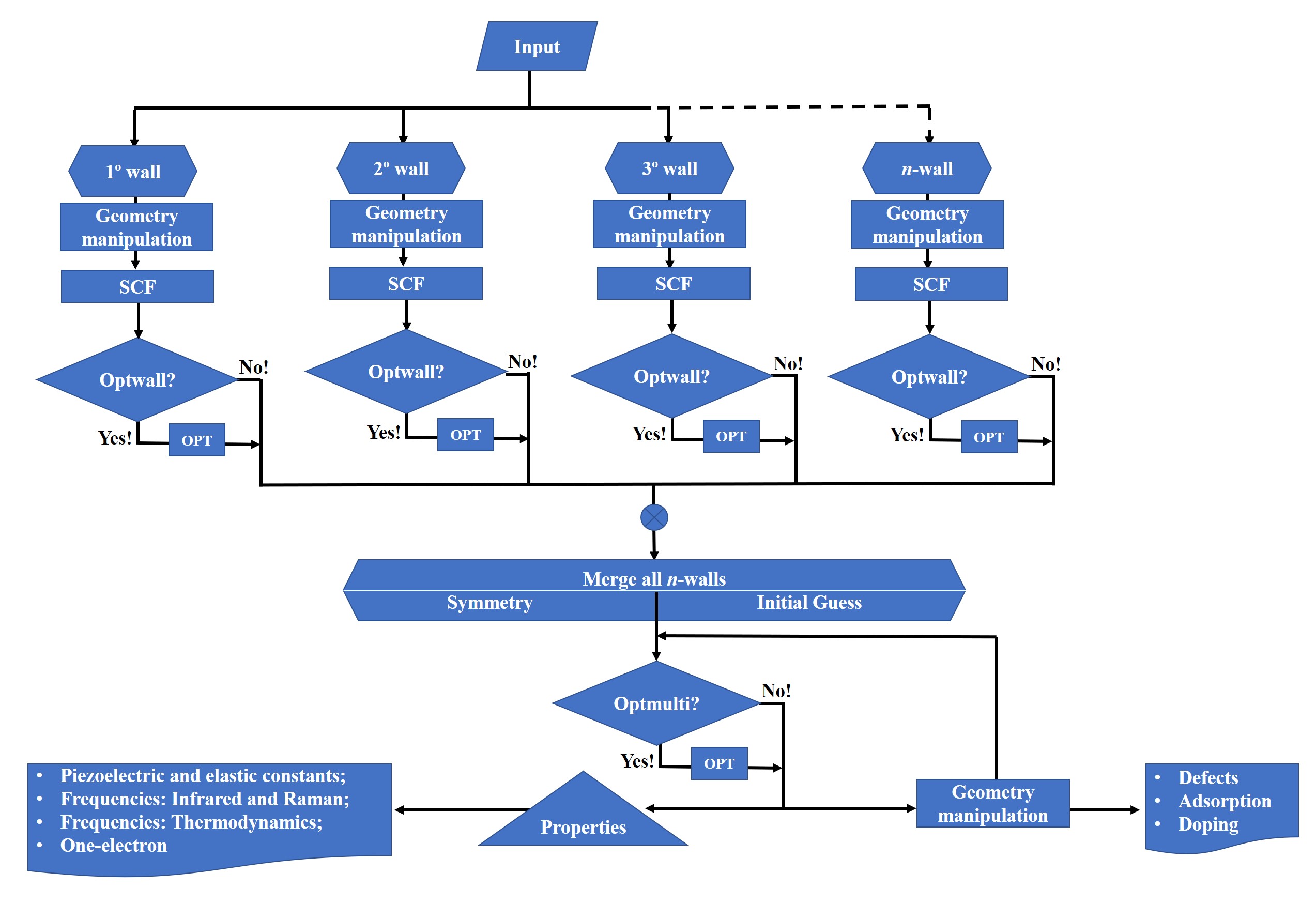

Supplement: Supplementary file 1 — jp1c01682_si_001.zip [file jp1c01682_si_001.zip › SI/figures_SI/Fig1_SI.jpg]

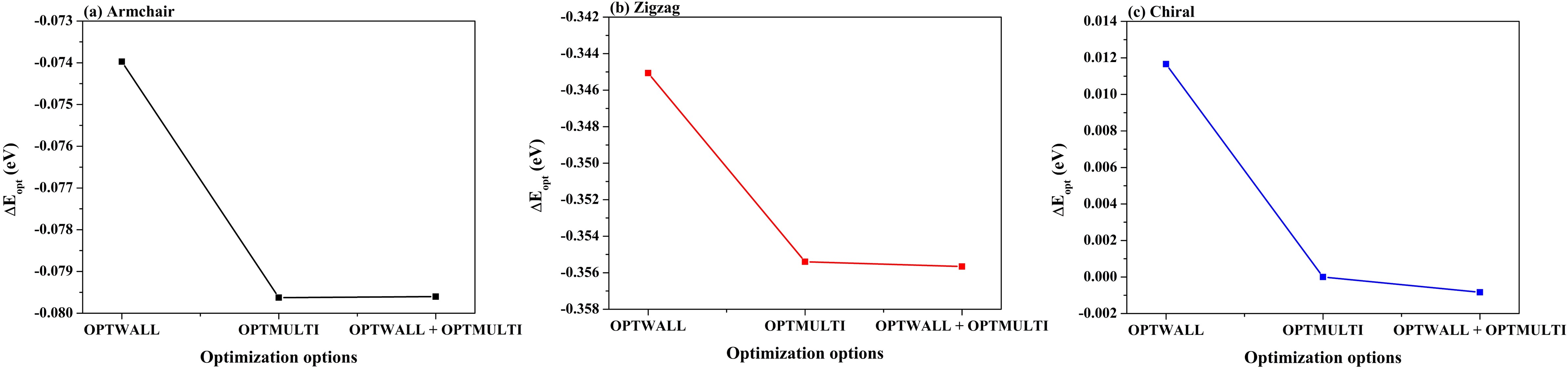

Supplement: Supplementary file 1 — jp1c01682_si_001.zip [file jp1c01682_si_001.zip › SI/figures_SI/Fig2_SI.jpg]

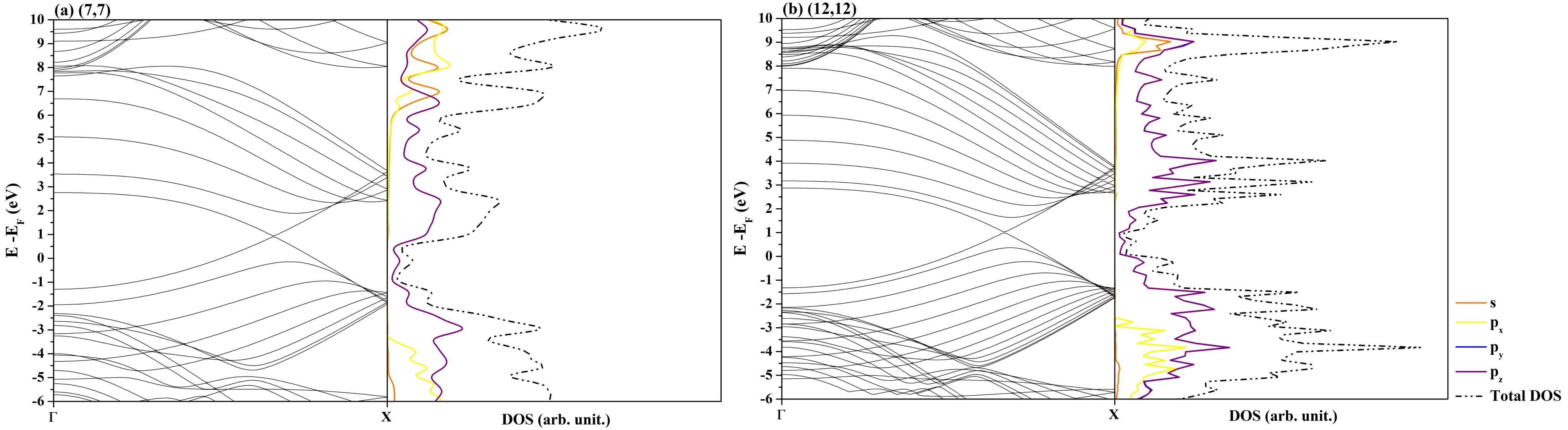

Supplement: Supplementary file 1 — jp1c01682_si_001.zip [file jp1c01682_si_001.zip › SI/figures_SI/Fig3a_SI.jpg]

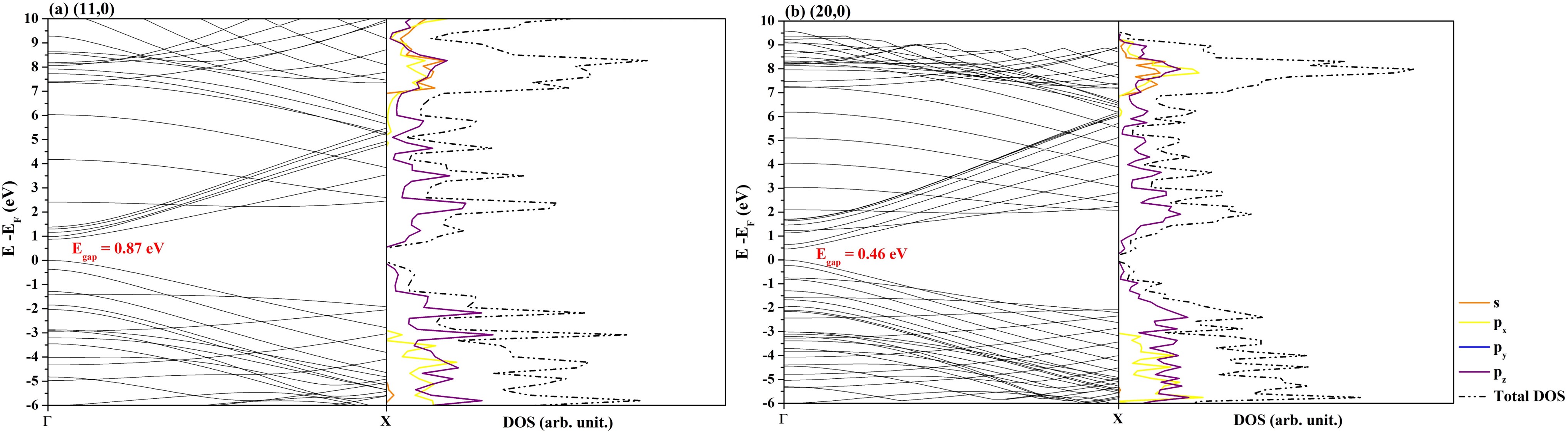

Supplement: Supplementary file 1 — jp1c01682_si_001.zip [file jp1c01682_si_001.zip › SI/figures_SI/Fig3b_SI.jpg]

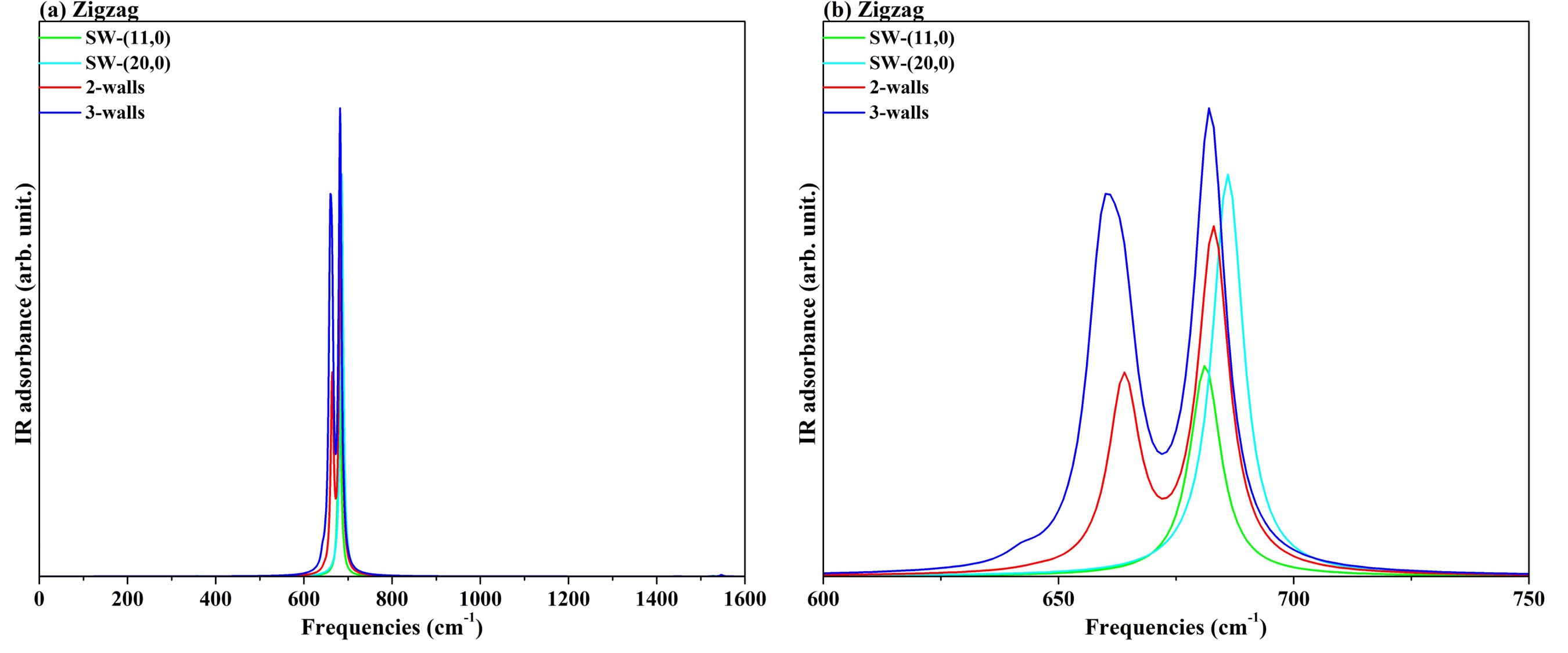

Supplement: Supplementary file 1 — jp1c01682_si_001.zip [file jp1c01682_si_001.zip › SI/figures_SI/Fig4_SI.jpg]

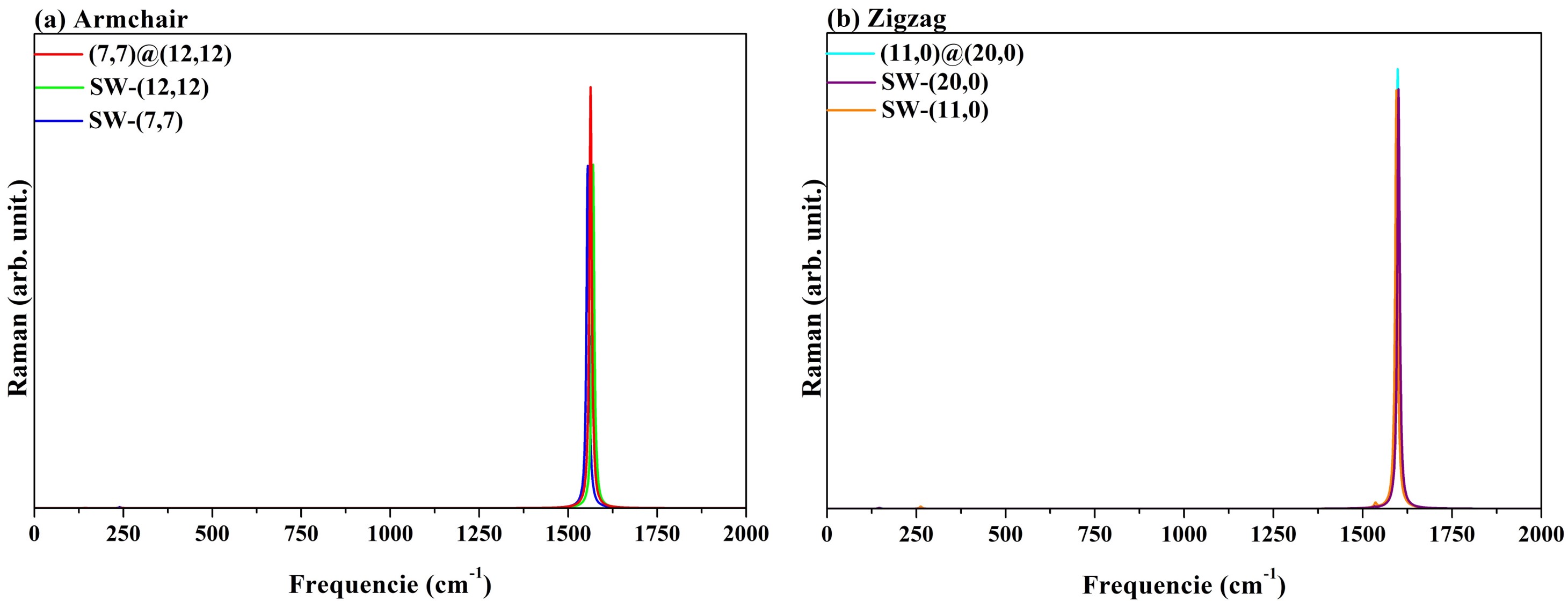

Supplement: Supplementary file 1 — jp1c01682_si_001.zip [file jp1c01682_si_001.zip › SI/figures_SI/Fig5_SI.jpg]
